# Supplementary material for: Functional roles of multiple Ton complex genes in a Sphingobium degrader of lignin-derived aromatic compounds
Source: Sci Rep. 2021 Nov 17;11:22444. doi: 10.1038/s41598-021-01756-8 (PMC8599685; doi:10.1038/s41598-021-01756-8)
Supplement: Supplementary file 1 — Supplementary Information. [file 41598_2021_1756_MOESM1_ESM.pdf]

Supplementary information

Functional roles of multiple Ton complex genes in a *Sphingobium* degrader of lignin-derived aromatic compounds

Masaya Fujita[1,4], Shodai Yano[1], Koki Shibata[1], Mizuki Kondo[2], Shojiro Hishiyama[3], Naofumi Kamimura[1], and Eiji Masai[1,*]

[1]*Department of Bioengineering, Nagaoka University of Technology, Nagaoka, Niigata, Japan*
[2]*Center for Integrated Technology Support, Nagaoka University of Technology, Nagaoka, Niigata, Japan*
[3]*Forestry and Forest Products Research Institute, Tsukuba, Ibaraki, Japan*
[4]*Present address: Structural Biology Research Center, Institute of Materials Structure Science, High Energy Accelerator Research Organization (KEK), Tsukuba, Ibaraki, Japan*

*Corresponding author:
Eiji Masai
Department of Bioengineering, Nagaoka University of Technology, Nagaoka, Niigata 940-2188, Japan
E-mail: emasai@vos.nagaokaut.ac.jp
TEL: +81 258 47 9428
ORCID: 0000-0001-9194-7483

Contents list

Supplementary tables: Table S1-S4
Supplementary figures: Fig. S1-S16
References

Table S1. Conservation of the *tonB1* operon genes of *Sphingobium* sp. SYK-6 in selected Sphingomonadaceae strains

Species and strain	*tonB1* operon	TonB1		ExbB1		ExbD1		ExbD2	
		Accession no.[a]	Sequence identity (%)[b]	Accession no.[a]	Sequence identity (%)[b]	Accession no.[a]	Sequence identity (%)[b]	Accession no.[a]	Sequence identity (%)[b]
Novosphingobium aromaticivorans DSM12444	+	ABD24466	47	ABD24465	66	ABD24464	43	ABD24463	56
Novosphingobium pentaromativorans US6-1	+	AIT80170	46	AIT80171	65	AIT80172	43	AIT80173	55
Novosphingobium resinovorum SA1	+	AOR77983	43	AOR77984	64	AOR77985	40	AOR77986	55
Novosphingobium sp. PP1Y	+	CCA94311	47	CCA94312	65	CCA94313	43	CCA94314	55
Sphingobium chlorophenolicum L-1	+	AEG50516	58	AEG50517	80	AEG50518	76	AEG50519	75
Sphingobium japonicum UT26S	+	BAI95564	58	BAI95565	81	BAI95566	75	BAI95567	74
Sphingobium sp. YBL2	+	AJR24448	58	AJR24449	80	AJR24450	73	AJR24451	75
Sphingomonas wittichii RW1	+	ABQ66489	44	ABQ66490	69	ABQ66491	67	ABQ66492	65
Blastomonas natatoria DSM 3183	+	PXW69510	52	PXW69511	71	PXW69512	55	PXW69513	66
Novosphingobium nitrogenifigens DSM 19370	+	EGD60281	43	EGD60282	35	EGD60284	48	EGD60285	60
Sphingobium yanoikuyae ATCC 51230	+	EKU75209	59	EKU75210	79	EKU75211	76	EKU75212	72
Sphingopyxis alaskensis RB2256	+	ABF52528	52	ABF52527	62	ABF52526	60	ABF52525	68

[a]Most similar proteins were searched using the BLAST-P program[1] showing the E-value is less than 1e-20.

[b]Amino acid sequence identity was calculated by the EMBOSS Needle pairwise alignment program.

Table S2. Conservation of the Tol-Pal system genes of *Sphingobium* sp. SYK-6 in selected Sphingomonadaceae and other bacterial strains

Species and strain	Tol-Pal cluster	ExbB2/TolQ		ExbD3/TolR		TolA		TolB		Pal	
		Accession no [a]	Sequence identity (%)[b]	Accession no.[a]	Sequence identity (%)[b]	Accession no.[a]	Sequence identity (%)[b]	Accession no[a]	Sequence identity (%)[b]	Accession no.[a]	Sequence identity (%)[b]
Novosphingobium aromaticivorans DSM12444	+	ABD25459	58	ABD25460	49	ABD25461	45	ABD25462	61	ABD25463	64
Novosphingobium pentaromativorans US6-1	+	AIT80992	56	AIT80993	54	AIT80994	40	AIT80995	58	AIT80996	64
Novosphingobium resinovorum SA1	+	AOR78292	56	AOR78293	50	AOR78294	36	AOR78295	59	AOR78296	64
Novosphingobium sp. PP1Y	+	CCA91793	56	CCA91794	48	CCA91795	37	CCA91796	58	CCA91797	64
Sphingobium chlorophenolicum L-1	+	AEG49724	62	AEG49725	59	AEG49726	48	AEG49727	70	AEG49728	70
Sphingobium japonicum UT26S	+	BAI98251	63	BAI98252	61	BAI98253	47	BAI98254	68	BAI98255	66
Sphingobium sp. YBL2	+	AJR22959	63	AJR22960	62	AJR22961	47	AJR22962	70	AJR22963	66
Sphingomonas wittichii RW1	+	ABQ68495	59	ABQ68494	54	ABQ68493	37	ABQ68492	59	ABQ68491	63
Blastomonas natatoria DSM 3183	+	PXW74505	61	PXW74504	58	PXW74503	40	PXW74502	66	PXW74501	59
Novosphingobium nitrogenifigens DSM 19370	+	EGD57947	58	EGD57948	46	EGD57949	38	EGD57950	59	EGD57951	64
Sphingobium yanoikuyae ATCC 51230	+	EKU74714	63	EKU74713	61	EKU74712	52	EKU74711	73	EKU74710	64
Sphingopyxis alaskensis RB2256	+	ABF52054	59	ABF52053	54	ABF52052	33	ABF52051	63	ABF52050	57
Escherichia coli K-12	+	P0ABU9	33	P0ABV6	26	P19934	13	P0A855	27	P0A912	34
Pseudomonas aeruginosa PAO1	+	P50598	36	P50599	35	P50600	20	P50601	30	Q9I4Z4	38
Caulobacter crescentus NA1000	+	ACL96805	39	ACL96804	39	ACL96803	30	ACL96802	45	ACL96801	44

[a]Most similar proteins were searched using the BLAST-P program[1] showing the E-value is less than 1e-10.

[b]Amino acid sequence identity was calculated by the EMBOSS Needle pairwise alignment program.

Table S3. Strains and plasmids used in this study

Strains or plasmids	Relevant characteristic(s)[a]	Reference or source
Strains		
Sphingobium sp.		
SYK-6	Wild type; Nal^r Sm^r	2
SME057	SYK-6 derivative; ΔSLG_38320 (*ompW*); Nal^r Sm^r	3
SME097	SYK-6 derivative; ΔSLG_34540 (*tonB2*); Nal^r Sm^r	3
SME291	SYK-6 derivative; ΔSLG_10800 (*exbB3*); Nal^r Sm^r	This study
SME295	SYK-6 derivative; ΔSLG_02490 (*exbD3*/*tolR*); Nal^r Sm^r	This study
SME296	SYK-6 derivative; ΔSLG_02500 (*exbB2*/*tolQ*); Nal^r Sm^r	This study
SME303	SYK-6 derivative; Δ*tonB3456*; Nal^r Sm^r	3
SME304	SYK-6 derivative; Δ*tonB23456*; Nal^r Sm^r	3
SME352	SYK-6 derivative; Δ *exbD3*/*tolR* and *ligI*; Nal^r Sm^r	This study
Escherichia coli		
HB101	*recA13 supE44 hsd20 ara-14 proA2 lacY1 galK2 rpsL20 xyl-5 mtl-1*	4
NEB 10-beta	*araD*139 Δ(*ara-leu*)7697 *fhuA lacX74 galK* (ϕ80 Δ*lacZ* Δ*M15*) *recA1 endA1 nupG rpsL* (Sm^r) Δ(*mrr-hsdRMS-mcrBC*)	New England Biolabs
Plasmids		
pRK2013	Tra^+ Mob^+ ColE1 replicon; Km^r	5
pJB861	RK2 ori broad-host-range expression vector; Km^r P_m *xylS*	6
pAK405	Plasmid for allelic exchange and markerless gene deletions in Sphingomonads; Km^r	7
pSEVA225	RK2 ori *lacZ* promoter probe broad host range vector; Km^r	8
pSEVA338	pBBR1 ori broad-host-range expression vector; Cm^r P_m *xylS*	8
pAK-02490	pAK405 with a 1.9-kb deletion cassette carrying up- and downstream regions of *tolR*/*exbD3*	This study
pAK-02500	pAK405 with a 2.0-kb deletion cassette carrying up- and downstream regions of *tolQ*/*exbB2*	This study
pAK-10800	pAK405 with a 2.0-kb deletion cassette carrying up- and downstream regions of *exbB3*	This study
pAK-ligI	pAK405 with a 2.0-kb deletion cassette carrying up- and downstream regions of *ligI*	9
pS-XR	pSEVA225 with a 0.8-kb PCR amplicon carrying *ddvR* and *ligXa* promoter regions	10
pS-tonB1	pSEVA338 with a 0.7-kb fragment carrying *tonB1*	3
pJB-tonB1	pJB861 with a 0.7-kb NotI-SacI fragment carrying *tonB1* from pS-tonB1	3
pS-t1-D1	pSEVA338 with a 2.1-kb fragment carrying *tonB1*, *exbB1*, and *exbD1*	3
pJB-t1-D1	pJB861 with a 2.1-kb NotI-SacI fragment carrying *tonB1*, *exbB1*, and *exbD1* from pS-t1-D1	3
pS-t1-D12	pSEVA338 with a 2.6-kb fragment carrying *tonB1*, *exbB1*, *exbD1*, and *exbD2*	3
pJB-t1-D12	pJB861 with a 2.6-kb NotI-SacI fragment carrying *tonB1*, *exbB1*, *exbD1*, and *exbD2* from pS-t1-D12	3
pJB-exbB1	pJB861 with a 0.8-kb fragment carrying *exbB1*	This study
pJB-exbD12	pJB861 with a 1.0-kb fragment carrying *exbD1* and *exbD2*	This study
pJB-*exbB2*/*tolQ*	pJB861 with a 0.7-kb fragment carrying *exbB2*/*tolQ*	This study
pJB-*exbD3*/*tolR*	pJB861 with a 0.5-kb fragment carrying *exbD3*/*tolR*	This study

[a]Nal^r, Sm^r, Km^r, and Cm^r, resistance to nalidixic acid, streptomycin, kanamycin, and chloramphenicol, respectively.

Table S4. Primers used in this study

Target gene	Primer	Sequences (5′ to 3′)
For gene disruption		
pAK-02490	Dis_TopF	CGGTACCCGGGGATCGCGTCTCGACATTCTGCT
(*exbD3*/*tolR*)	Dis_TopR	GTGTGATGGGCGAACTGA
	Dis_BotF	TCAGTTCGCCCATCACACGCGCTGTGACCATGAAGA
	Dis_BotR	CGACTCTAGAGGATCGGACGATGACCTGCTCGT
pAK-02500	Dis_TopF	CGGTACCCGGGGATCGCATCACTCTCCAGTCCC
(*exbB2*/*tolQ*)	Dis_TopR	GCCGTCATCGCCTACAAC
	Dis_BotF	GTTGTAGGCGATGACGGCGCCGAGCATGACGATCTT
	Dis_BotR	CGACTCTAGAGGATCTCGCCAGCCCGATGAAAA
pAK-10800	Dis_TopF	CGGTACCCGGGGATCTCGGCGTCTTCAATCCAC
(*exbB3*)	Dis_TopR	AGGACCGTCCAGAGAACC
	Dis_BotF	GGTTCTCTGGACGGTCCTGGTGGGCGTTGTCGCTTA
	Dis_BotR	CGACTCTAGAGGATCGTGGTGGAAAATCGACGG
For confirmation of gene disruption		
exbD3/*tolR*	exbD3_F	GAGCGCGACGTCGAAAAG
exbB2/*tolQ*	exbB2_F	TCAGTTCGCCCATCACACGCGCTGTGACCATGAAGA
exbB3	exbB3_F	AACTGGCCTCGCGCTATG
Construction of plasmids		
pJB-exbB1	Forward	GCCTAGGCCGCGGCCGCGCGCGGCCACTTCACTGACTGG
	Reverse	ATCCCCGGGTACCGAGCTCGTCAGGCCTTGGCAGCAGC
pJB-exbD12	Forward	GCCTAGGCCGCGGCCGCGCGGGCTACGGCCCAGAAGTAG
	Reverse	TACCGAGCTCGAATTTCAGAACACGCCGCCGTA
pJB-exbD3/tolR	Forward	TCACCATGGGAAGCTTCGTGCACCACGTTCAGCCGGGA
	Reverse	GAATTCCTGCAGGATATCTGCTATTGATCGCCAGATGAACGCG
pJB-exbB2/tolQ	Forward	TCACCATGGGAAGCTTCGTGTCAGACAAGCCCGGAGAG
	Reverse	GAATTCCTGCAGGATATCTGTCAGGCTTCAAGCTCCCG

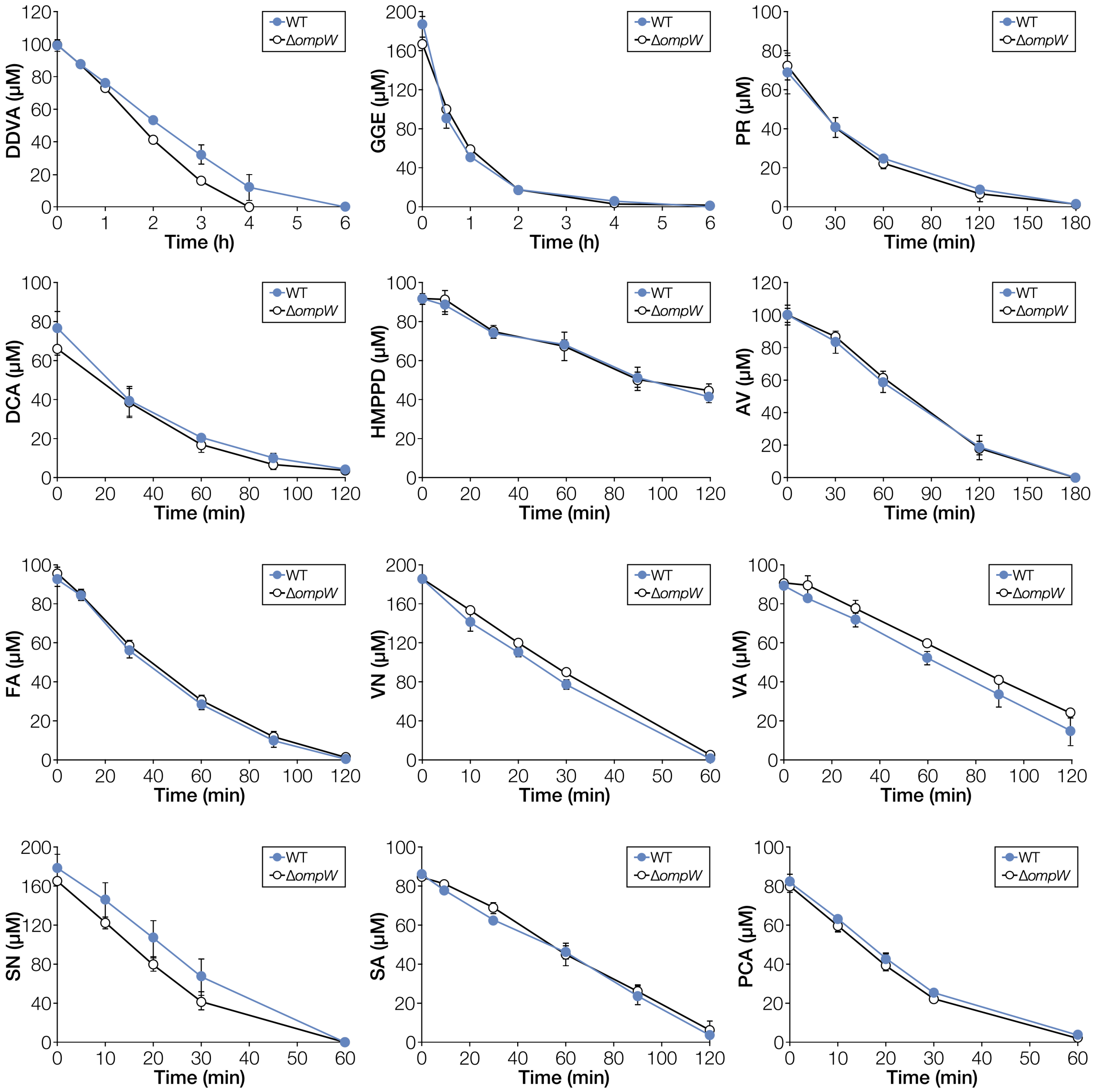

Fig. S1 Conversion of lignin-derived aromatic compounds by resting cells of Δ*ompW*. Cells of SYK-6 and Δ*ompW* were incubated with 100 μM DDVA, 200 μM GGE, 100 μM PR, 100 μM DCA, 100 μM HMPPD, 100 μM AV, 100 μM FA, 200 μM VN, 100 μM VA, 200 μM SN, 100 μM SA, and 100 μM PCA, respectively. Portions of the reaction mixtures were collected, and the amount of substrate was measured using HPLC. Each value is the average ± the standard deviation of three independent experiments. This figure was generated using Canvas X Draw version 7.0.2. (https://www.canvasgfx.com/products/canvas-x-draw).

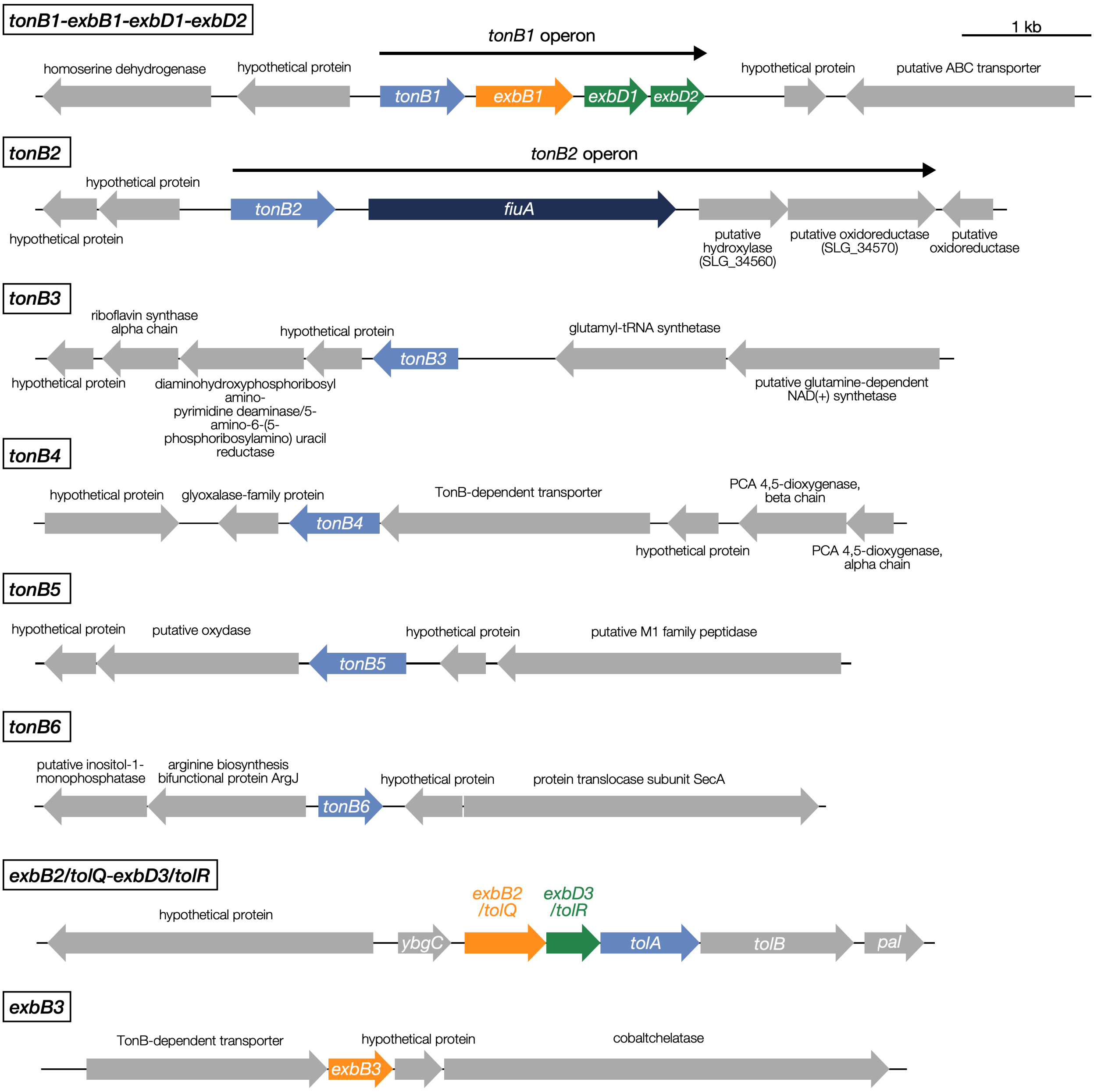

Fig. S2 Organization of the putative *tonB*, *exbB*, and *exbD* genes in the SYK-6 genome. *tonB1*–*exbB1*–*exbD1*–*exbD2* and *tonB2*–*fiuA*–SLG_34560–SLG_34570 each form an operon[3,11]. This figure was generated using Canvas X Draw version 7.0.2. (https://www.canvasgfx.com/products/canvas-x-draw).

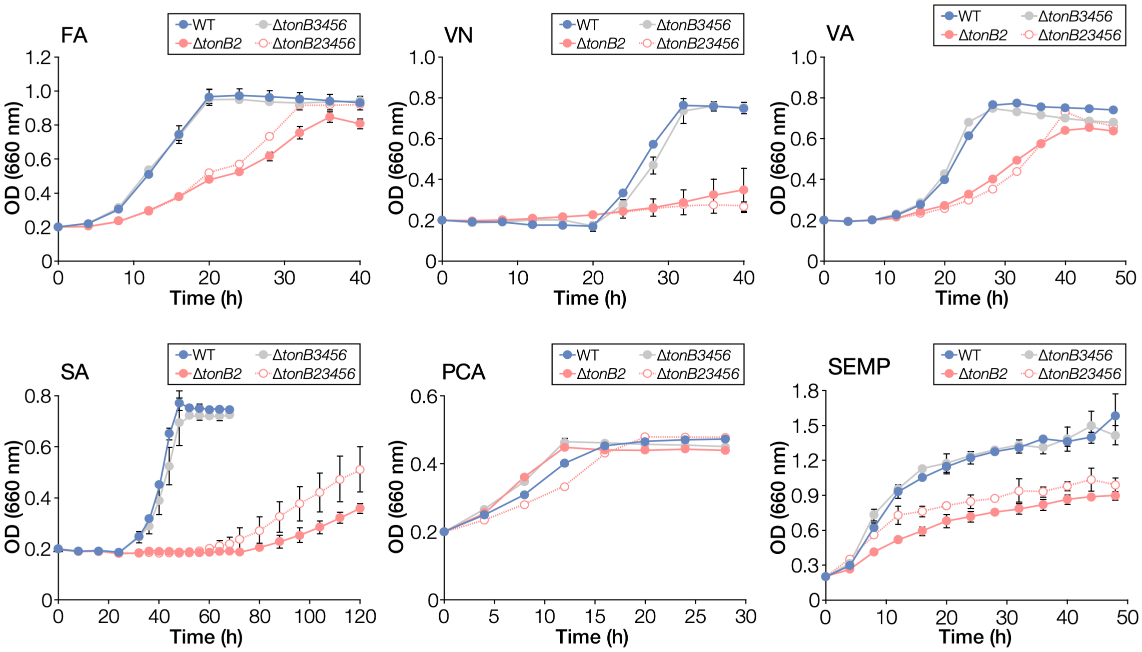

Fig. S3 Growth of *tonB* multiple mutants on lignin-derived aromatic compounds. Cells of SYK-6, Δ*tonB2*, Δ*tonB3456*, and Δ*tonB23456* were incubated in Wx medium containing 5 mM FA, VN, VA, SA, or PCA, and Wx medium containing SEMP. Cell growth was monitored by measuring the OD$_{660}$. Each value is the average ± the standard deviation of three independent experiments. The growth data on VA, PCA, and SEMP were reproduced from our previous paper (M. Fujita et al., *Sci. Rep*., 2020, 10, 12177, https://doi.org/10.1038/s41598-020-68984-2)[11]. This figure was generated using Canvas X Draw version 7.0.2. (https://www.canvasgfx.com/products/canvas-x-draw).

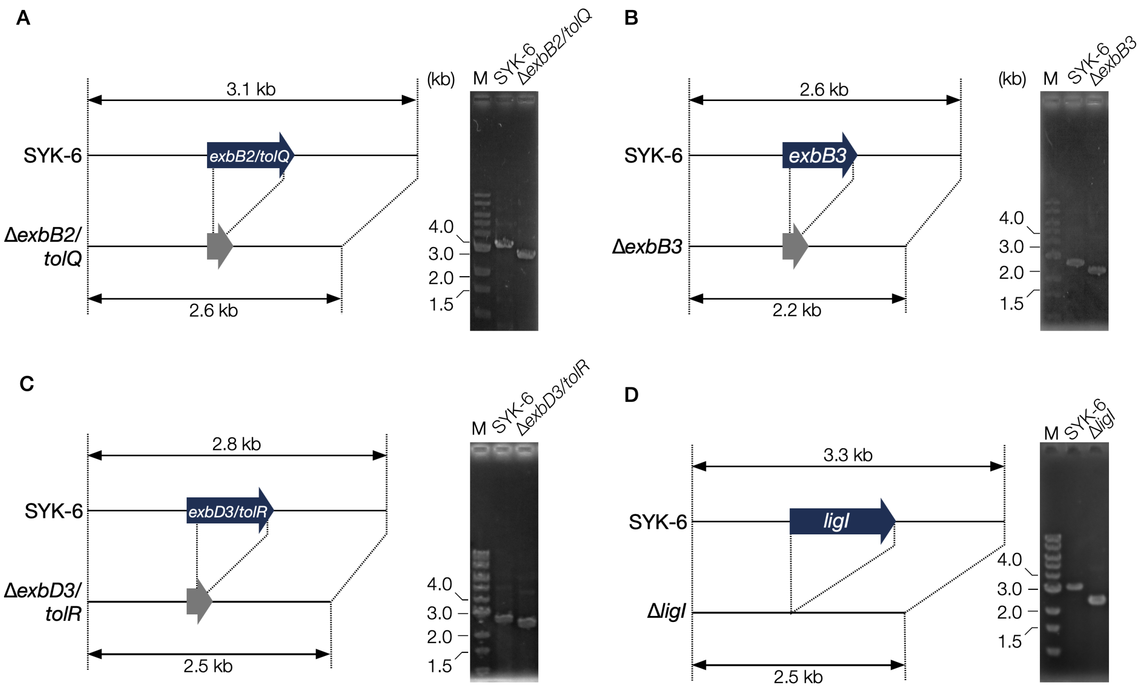

Fig. S4 Construction of mutants. (Left) Schematic representations of the disruption of *exbB2/tolQ* (A), *exbB3* (B), *exbD3/tolR* (C), and *ligI* (D). (Right) Colony PCR analysis of each mutant using the primer pairs shown in Table S4. M, molecular size markers. This figure was generated using Canvas X Draw version 7.0.2. (https://www.canvasgfx.com/products/canvas-x-draw).

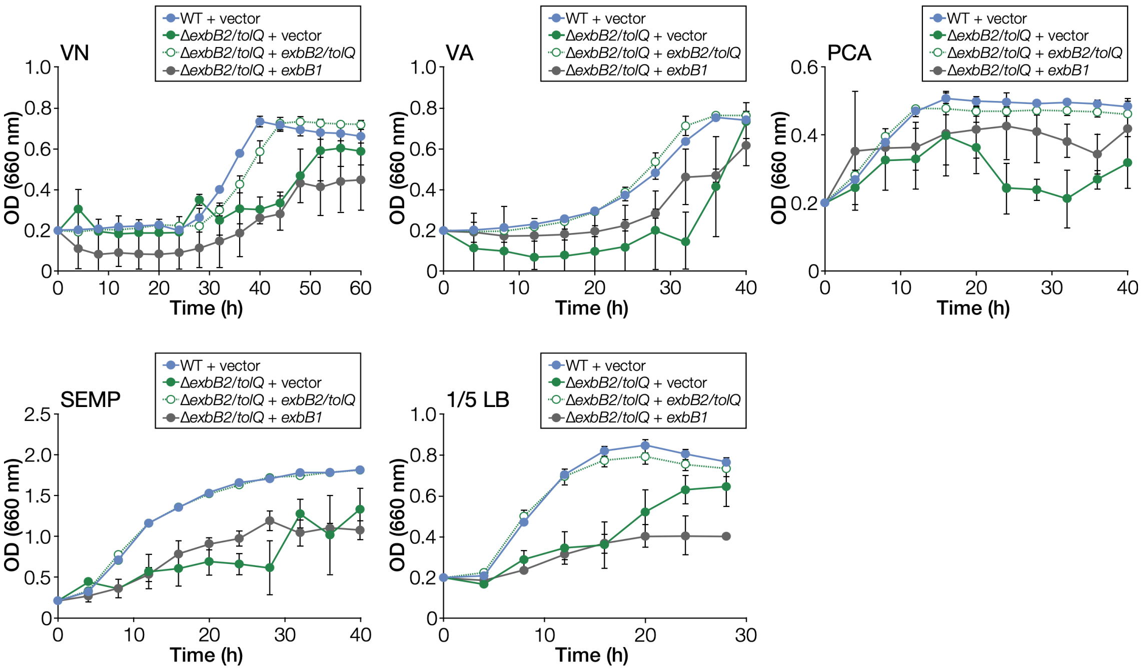

Fig. S5 Growth complementation of *exbB2*/*tolQ* mutant. Cells of SYK-6(pJB861), Δ*exbB2*/*tolQ*(pJB861), Δ*exbB2*/*tolQ*(pJB-exbB2/tolQ), and Δ*exbB2*/*tolQ*(pJB-exbB1) were incubated in Wx medium containing 1 mM *m*-toluate and 5 mM VN, VA, or PCA, Wx medium containing 1 mM *m*-toluate and SEMP, and diluted LB containing 1 mM *m*-toluate. Cell growth was monitored by measuring the OD_{660}. Each value is the average ± the standard deviation of three independent experiments. This figure was generated using Canvas X Draw version 7.0.2. (https://www.canvasgfx.com/products/canvas-x-draw).

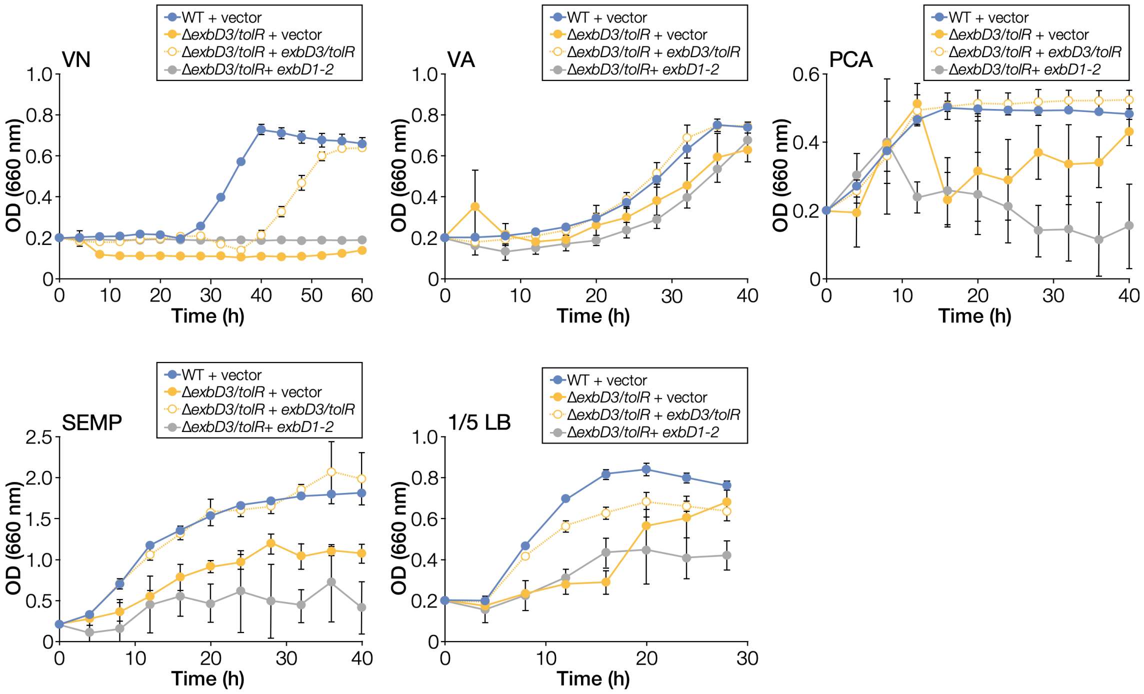

Fig. S6 Growth complementation of *exbD3/tolR* mutant. Cells of SYK-6(pJB861), Δ*exbD3*/*tolR*(pJB861), Δ*exbD3*/*tolR*(pJB-exbD3/tolR), and Δ*exbD3*/*tolR*(pJB-exbD12) were incubated in Wx medium containing 1 mM *m*-toluate and 5 mM VN, VA, or PCA, Wx medium containing 1 mM *m*-toluate and SEMP, and diluted LB containing 1 mM *m*-toluate. Cell growth was monitored by measuring the OD_{660}. Each value is the average ± the standard deviation of three independent experiments. This figure was generated using Canvas X Draw version 7.0.2. (https://www.canvasgfx.com/products/canvas-x-draw).

A

```
ExbB1_SYK-6      GLFGTVIGIYRALIKIGASGQASIDAVAGPVGEALIMTALGLAVAVPAVLAYNWLQRRNK 208
ExbB2/TolQ_SYK-6 GLFGTVWGIMRSFTAIAGEQNTSLAVVAPGIAEALFATAIGLFAAIPAVIAYNRLSHGVN 214
ExbB3_SYK-6      GLMGTLIPMATALSGLA---RGDLQILASNMVIAFSSTVVGLAVGVVA---YLVAMVREG 155
ExbB_E. coli     GLFGTVWGIMNSFIGIAQTQTTNLAVVAPGIAEALLATAIGLVAAIPAVVIYNVFARQIG 203
TolQ_E. coli     GLFGTVWGIMHAFIALGAVKQATLQMVAPGIAEALIATAIGLFAAIPAVMAYNRLNQRVN 200
                 **:**:  :  ::  :.      :  :*  :  *:  *.:** ..: *   *
```

B

```
ExbD1_SYK-6      ----MAMSAGGGGDDAPMSDINTTPLVDVMLVLLIIFLIAVPVVIQTVEVNLPKIAFEPT 56
ExbD2_SYK-6      -----MAMSGGRDDGEPMMEMNTTPLIDVMLVLLIMFIITIPIQTHAVKIDLPQNADAPQ 55
ExbD3/TolR_SYK-6 MSMSLPPGRRGGGRRAPMAEINVTPLVDVMLVLLIIFMVTAPLLVTGVPIRLPESRARAL 60
ExbD_E. coli     MAMHLNENL---DDNGEMHDINVTPFIDVMLVLLIIFMVAAPLATVDVKVNLPASTSTPQ 57
TolR_E. coli     -----MARARGRGRRDLKSEINIVPLLDVLLVLLLIFMATAPIITQSVEVDLPDATESQA 55
                             .      ::* .*::**:****::*: : *:    * : **
```

Fig. S7 Conserved amino acid residues between ExbB and TolQ and between ExbD and TolR. (A) T148, E176, and T181 (ExbB_*E. coli* numbering) are essential for ExbB[12]. (B) D25 (ExbD_*E. coli* numbering) is essential for ExbD[12]. Multiple alignments were constructed using the Clustal Omega program[13]. Accession numbers: ExbB_*E. coli*, P0ABU7; TolQ_*E. coli*, P0ABU9; ExbD_*E. coli*, P0ABV2; TolR_*E. coli*, P0ABV6. This figure was generated using Canvas X Draw version 7.0.2. (https://www.canvasgfx.com/products/canvas-x-draw).

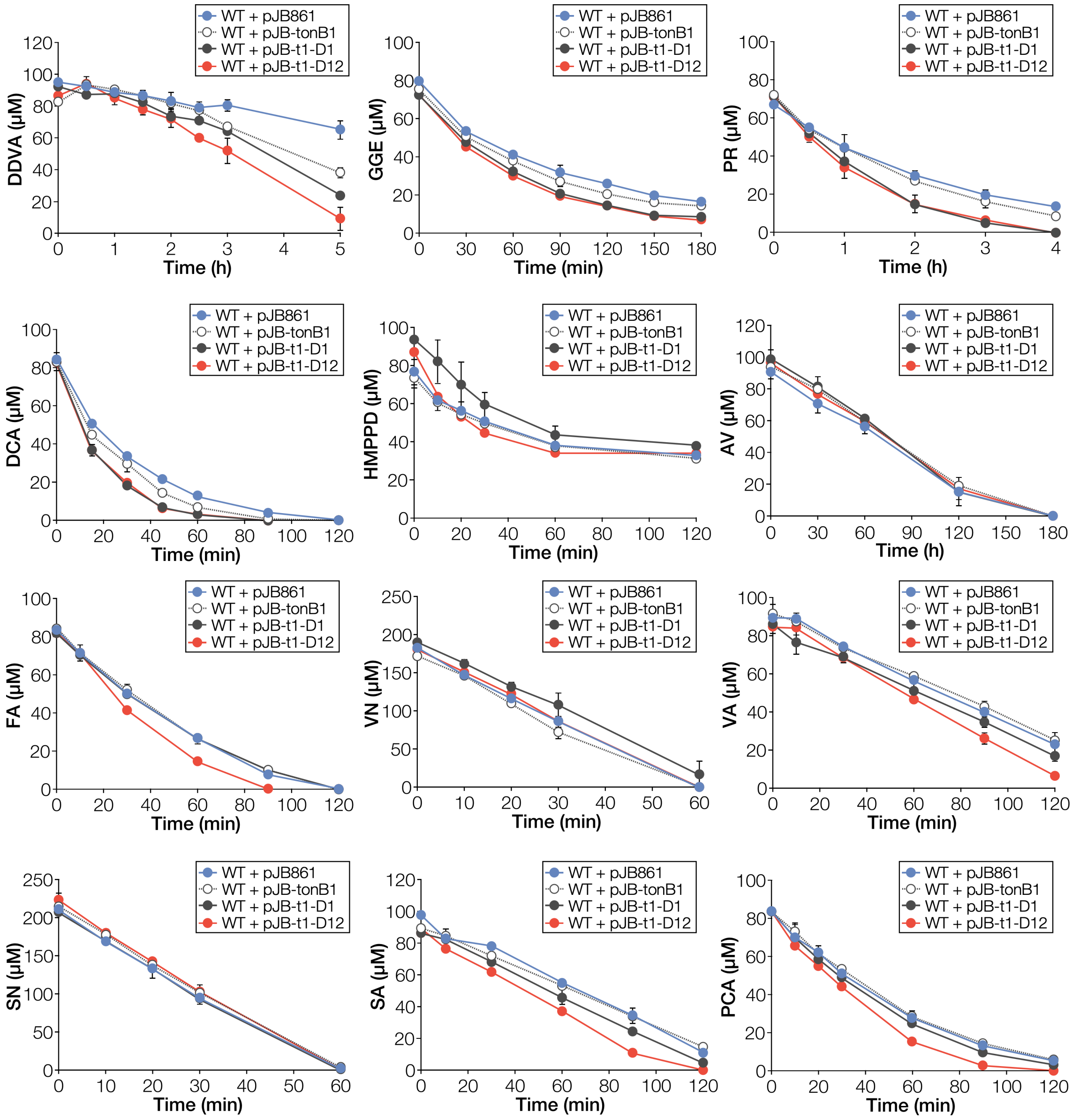

Fig. S8 Conversion of lignin-derived aromatic compounds by SYK-6 cells overexpressing the *tonB1* operon genes. Cells of SYK-6(pJB861), SYK-6(pJB-tonB1), SYK-6(pJB-t1-D1), and SYK-6(pJB-t1-D12) were incubated with 100 μM DDVA, 100 μM GGE, 100 μM PR, 100 μM DCA, 100 μM HMPPD, 100 μM AV, 100 μM FA, 200 μM VN, 100 μM VA, 200 μM SN, 100 μM SA, and 100 μM PCA, respectively. Portions of the reaction mixtures were collected, and the amount of substrate was measured using HPLC. Each value is the average ± the standard deviation of three independent experiments. This figure was generated using Canvas X Draw version 7.0.2. (https://www.canvasgfx.com/products/canvas-x-draw).

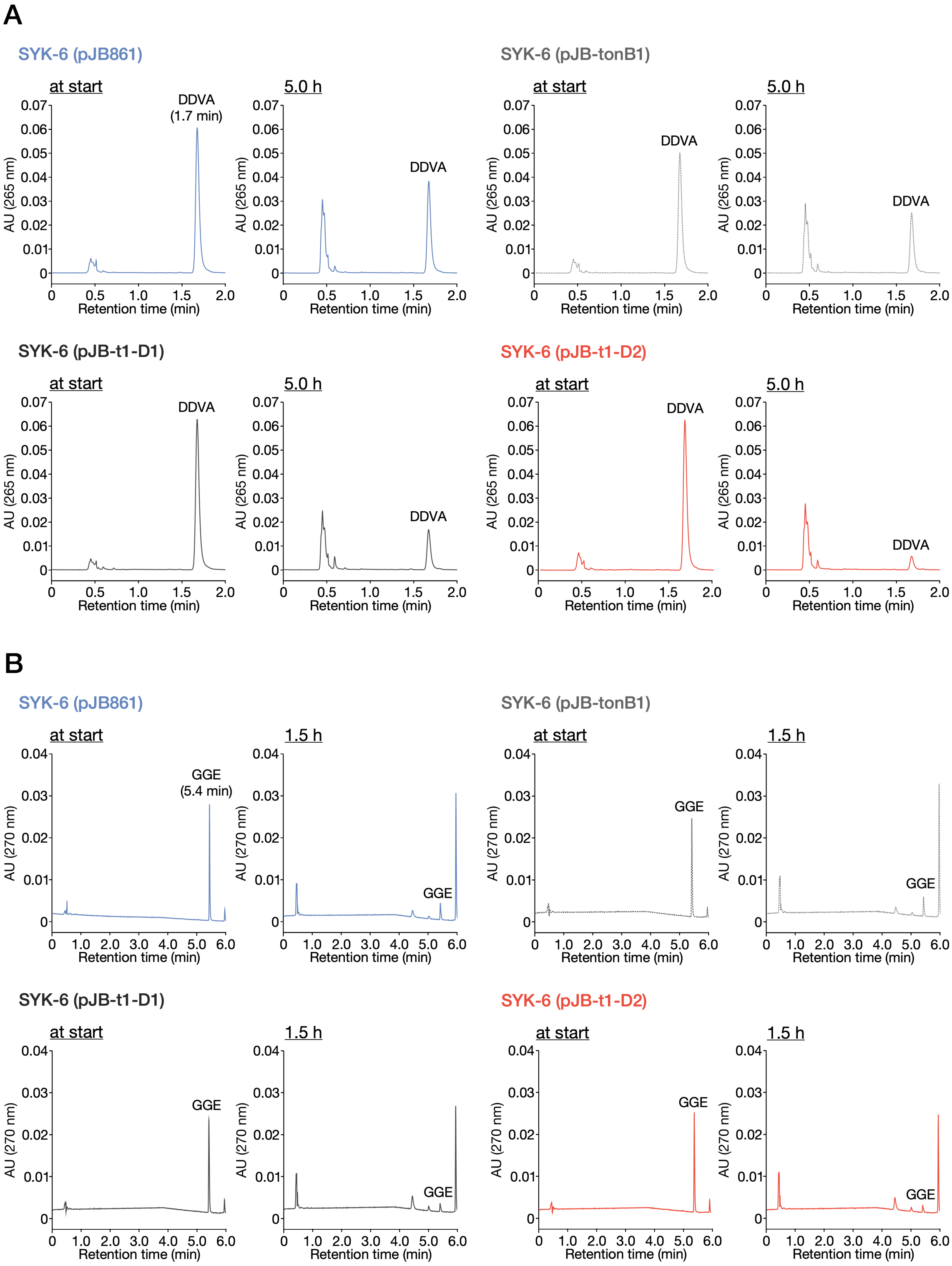

Fig. S9 HPLC chromatograms of conversion of lignin-derived aromatic compounds by SYK-6 cells overexpressing the *tonB1* operon genes. Conversion of DDVA (A; at start and 5.0 h), GGE (B; at start and 1.5 h), PR (C; at start and 2.0 h), DCA (D; at start and 0.5 h), HMPPD (E; at start and 0.5 h), AV (F;

at start and 2.0 h), FA (G; at start and 1.0 h), VN (H; at start and 0.5 h), VA (I; at start and 2.0 h), SN (J; at start and 0.5 h), SA (K; at start and 1.5 h), and PCA (L; at start and 1.0 h) by cells of SYK-6(pJB861), SYK-6(pJB-tonB1), SYK-6(pJB-t1-D1), and SYK-6(pJB-t1-D12). This figure was generated using Canvas X Draw version 7.0.2. (https://www.canvasgfx.com/products/canvas-x-draw).

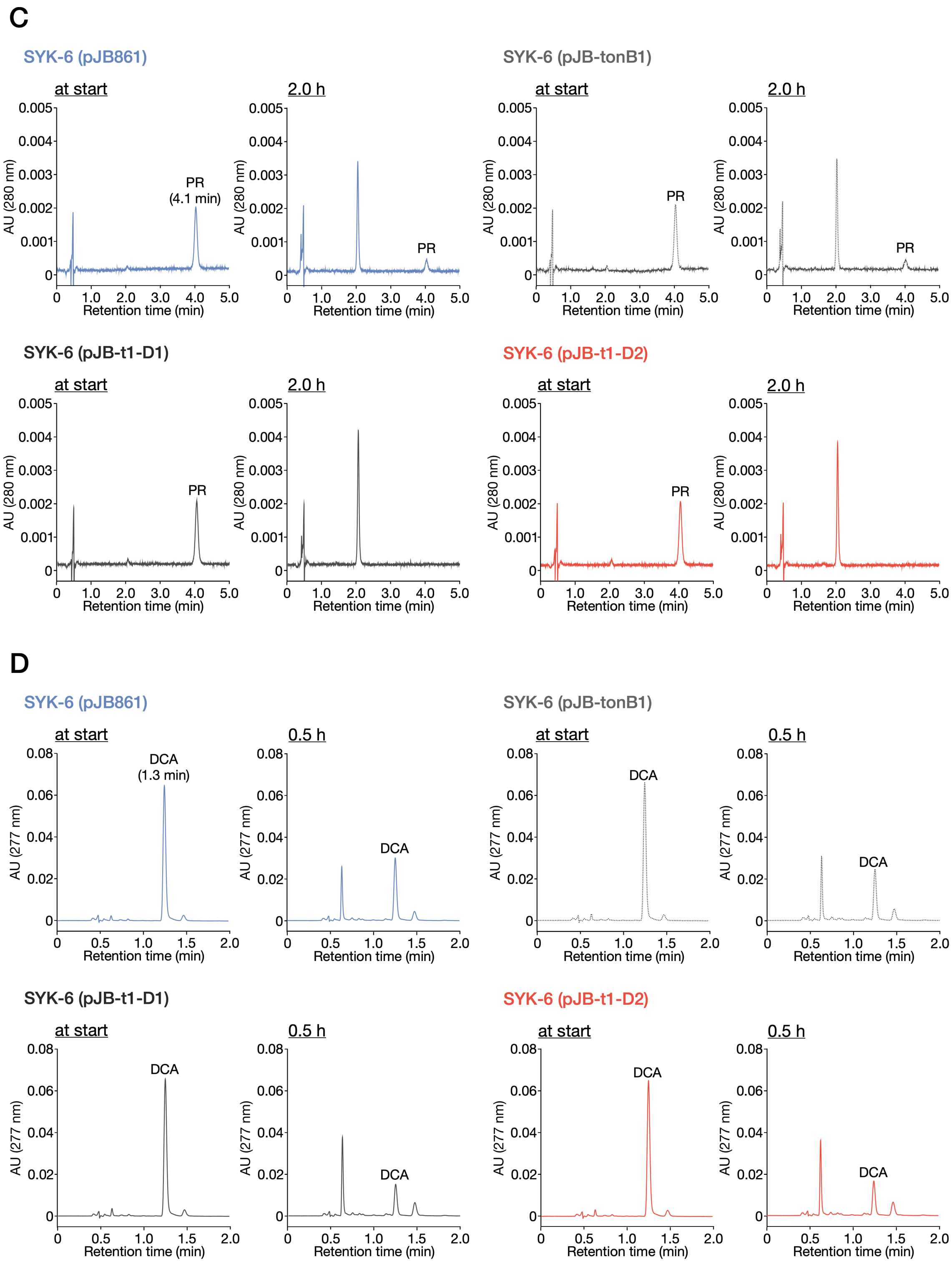

Fig. S9 –*continued.*

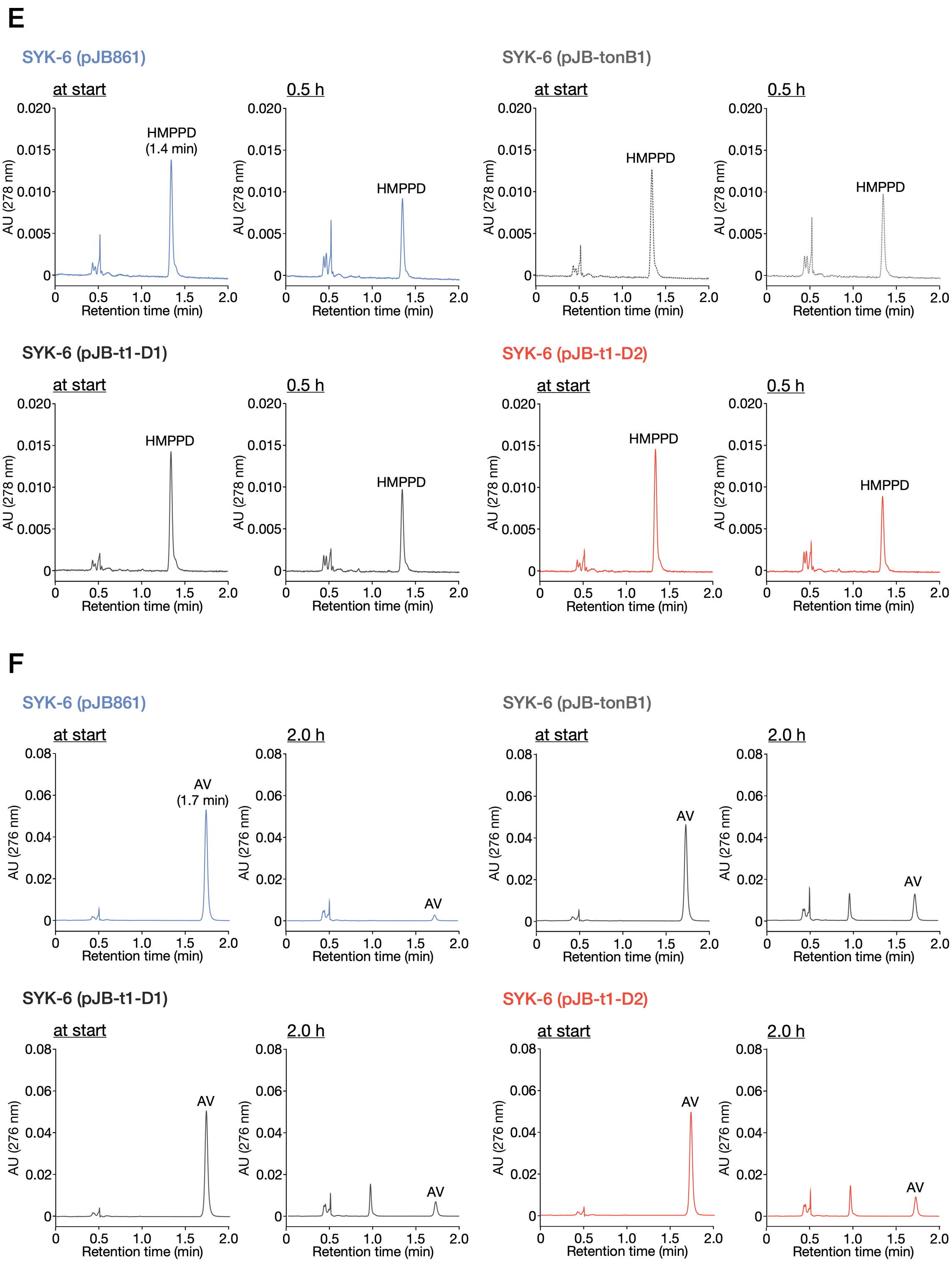

Fig. S9 –*continued.*

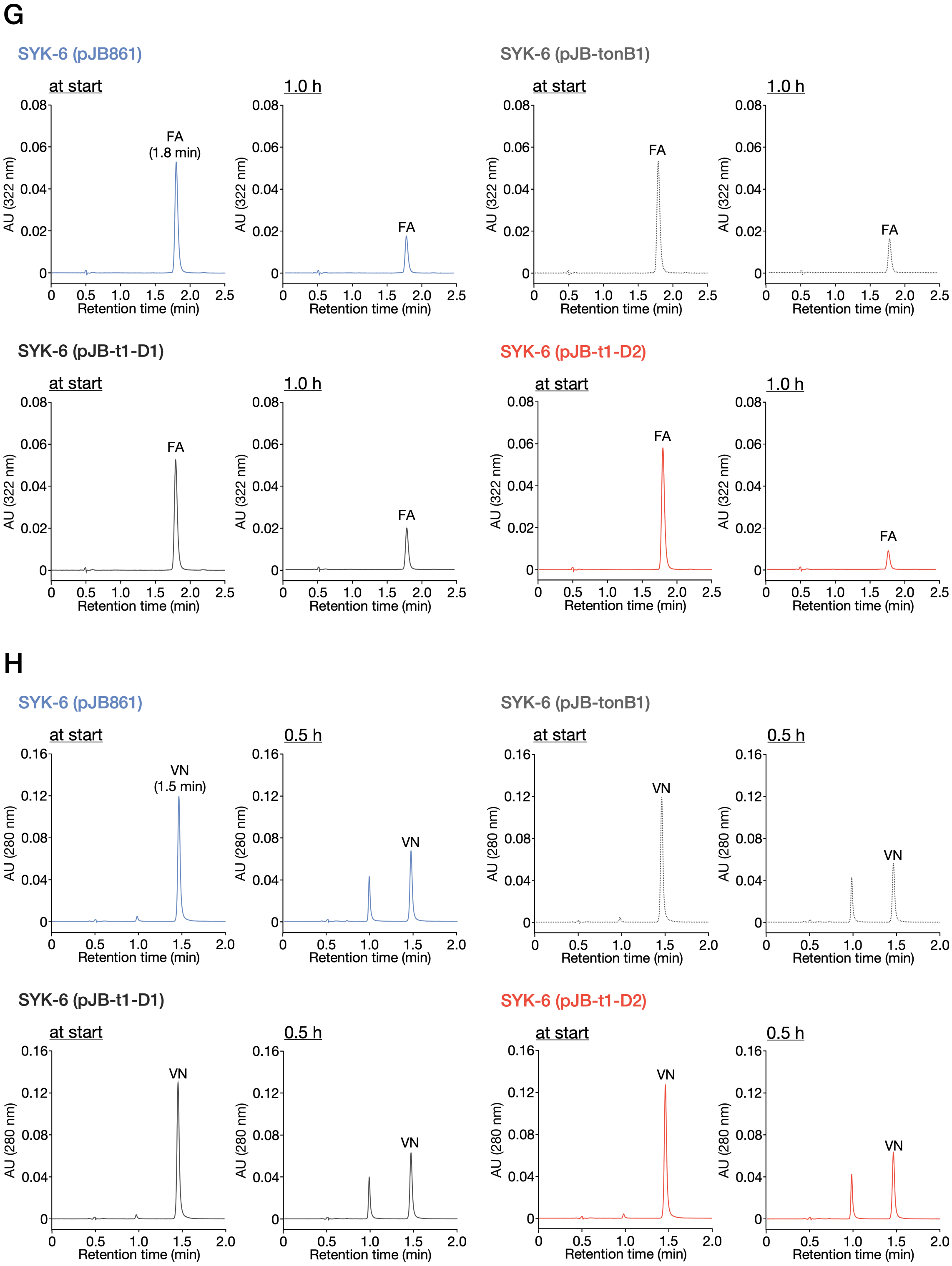

Fig. S9 –*continued.*

I

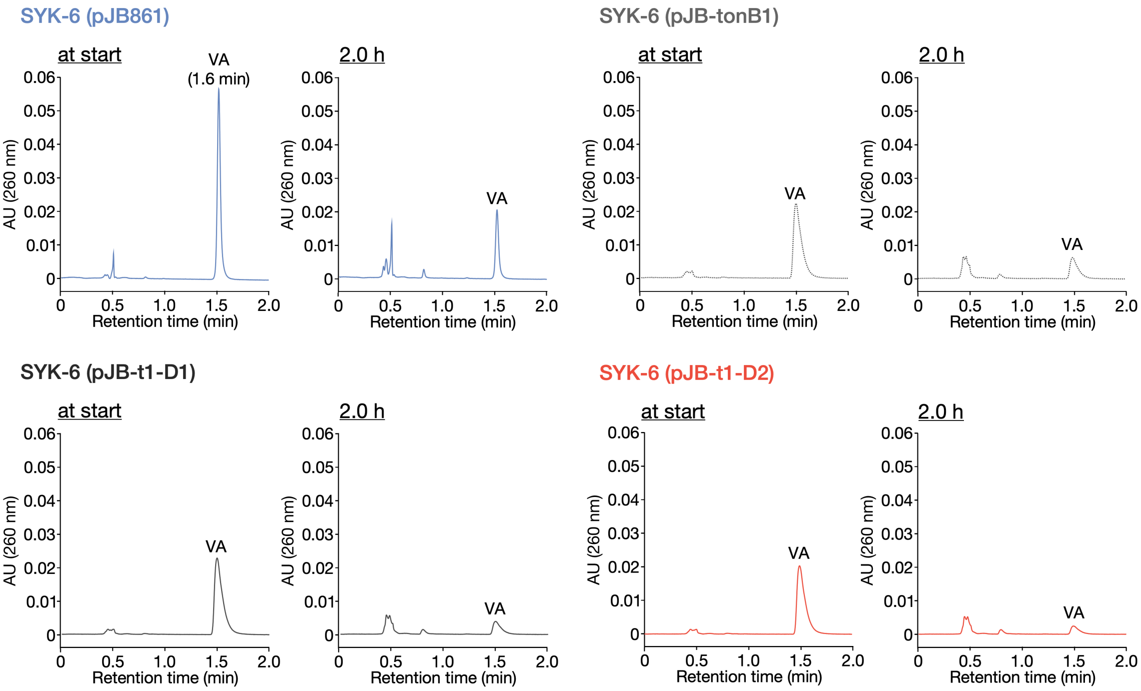

J

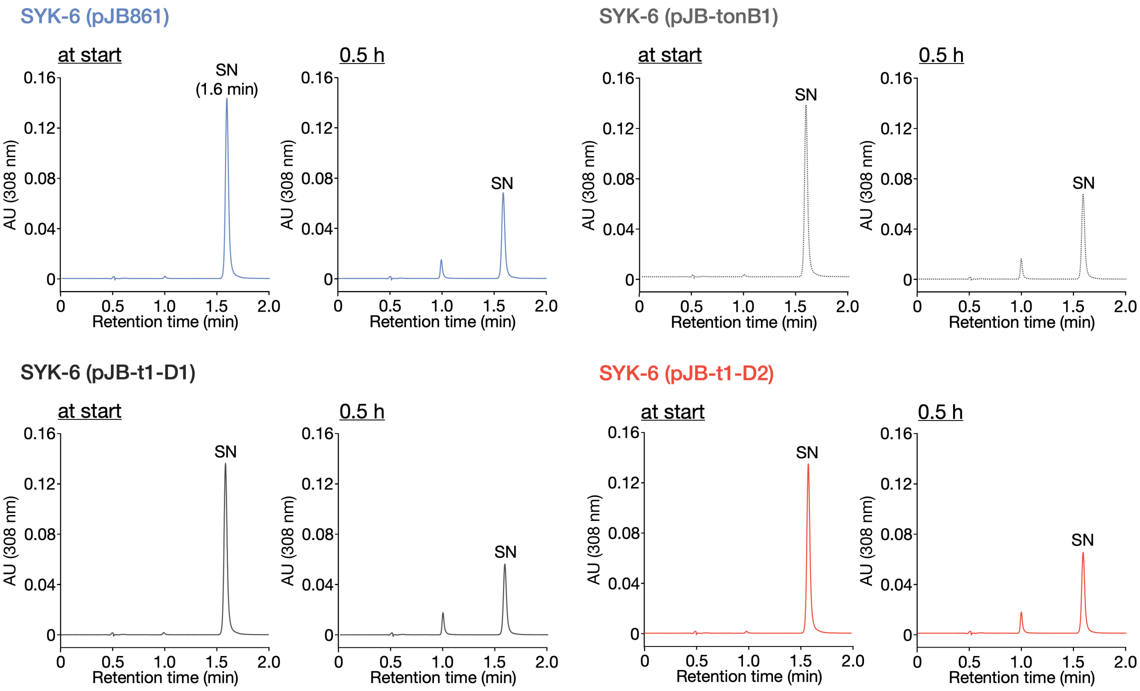

Fig. S9 –*continued.*

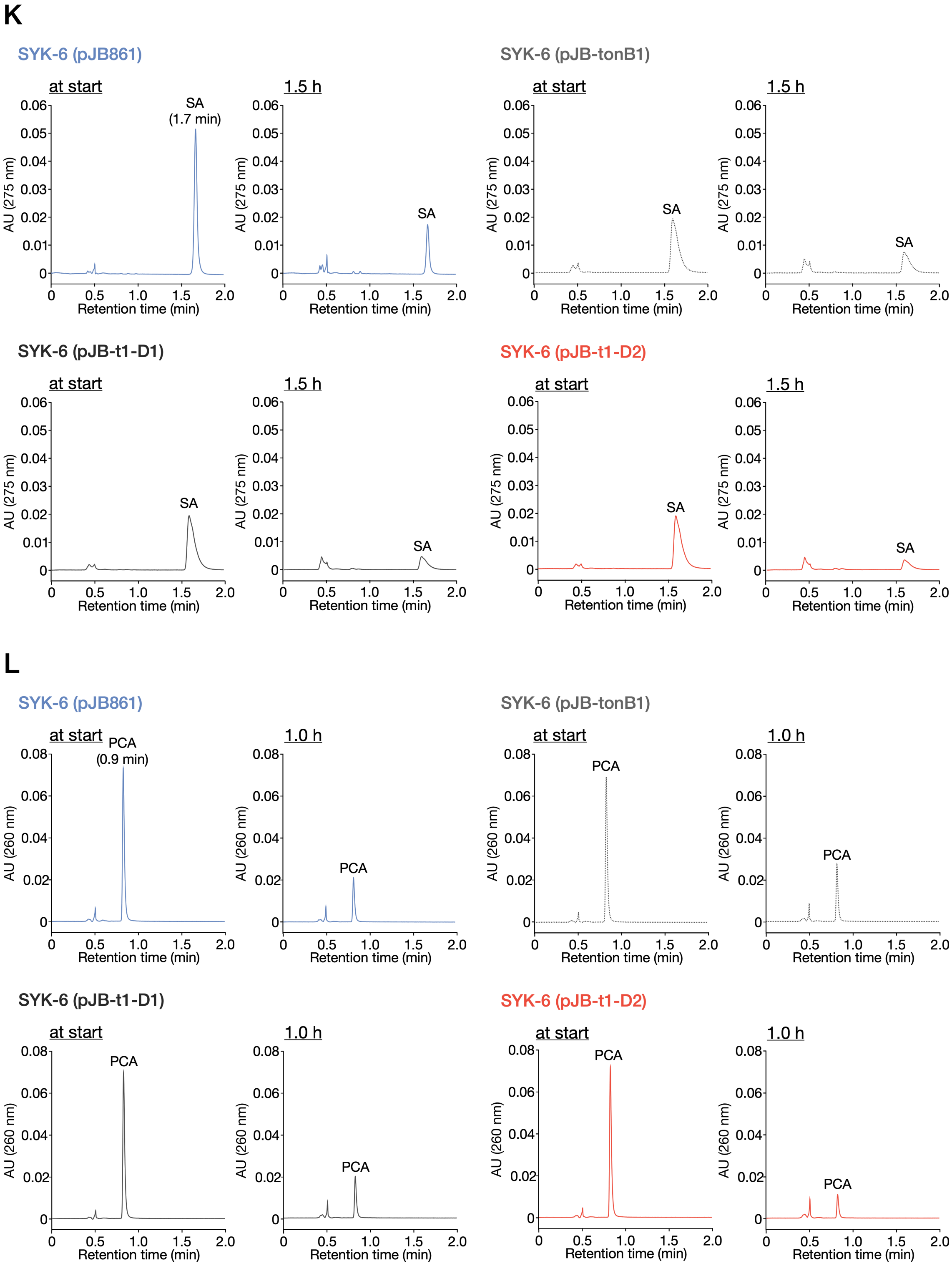

Fig. S9 –*continued.*

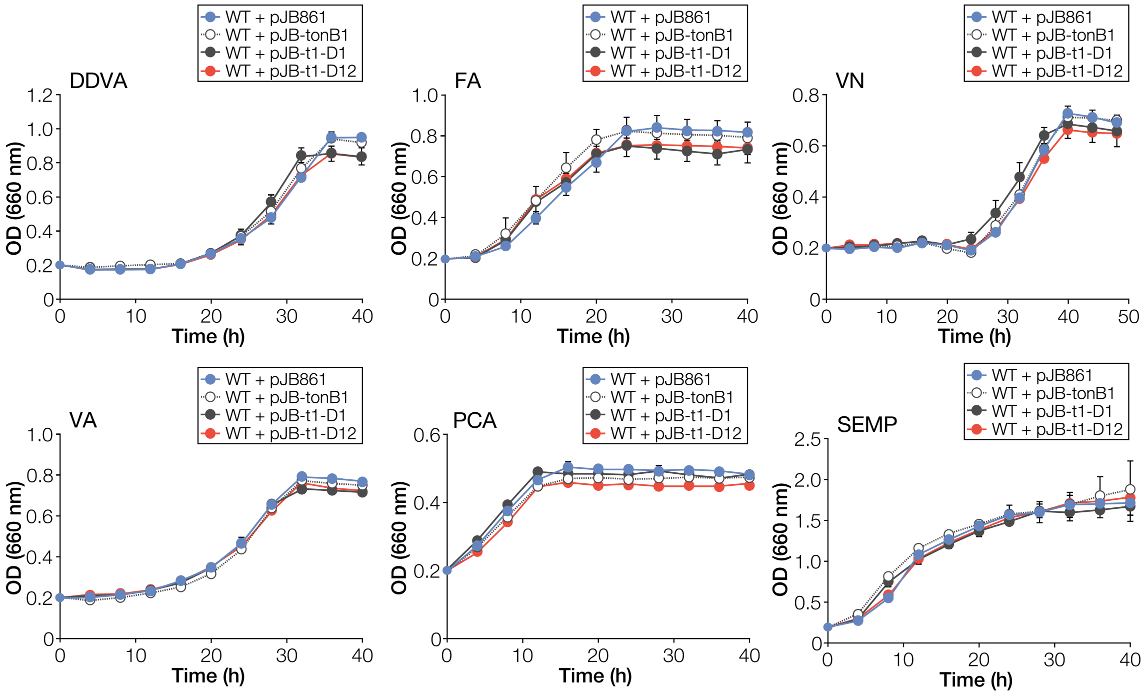

Fig. S10 Growth of SYK-6 cells overexpressing the *tonB1* operon genes. Cells of SYK-6(pJB861), SYK-6(pJB-tonB1), SYK-6(pJB-t1-D1), and SYK-6(pJB-t1-D12) were incubated in Wx medium containing 0.5 mM *m*-toluate and 5 mM DDVA, FA, VN, VA, or PCA and Wx medium containing 0.5 mM *m*-toluate and SEMP. Cell growth was monitored by measuring the OD_{660}. Each value is the average ± the standard deviation of three independent experiments. This figure was generated using Canvas X Draw version 7.0.2. (https://www.canvasgfx.com/products/canvas-x-draw).

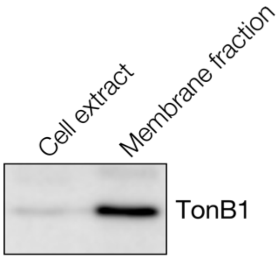

Fig. S11 Cellular localization of TonB1. Western blot analysis using anti-TonB1 antibodies was performed against the cell extract and total membrane fraction (10 μg protein each) obtained from SYK-6 cells grown in LB. The uncropped blot image is shown in Fig. S16. This figure was generated using Canvas X Draw version 7.0.2. (https://www.canvasgfx.com/products/canvas-x-draw).

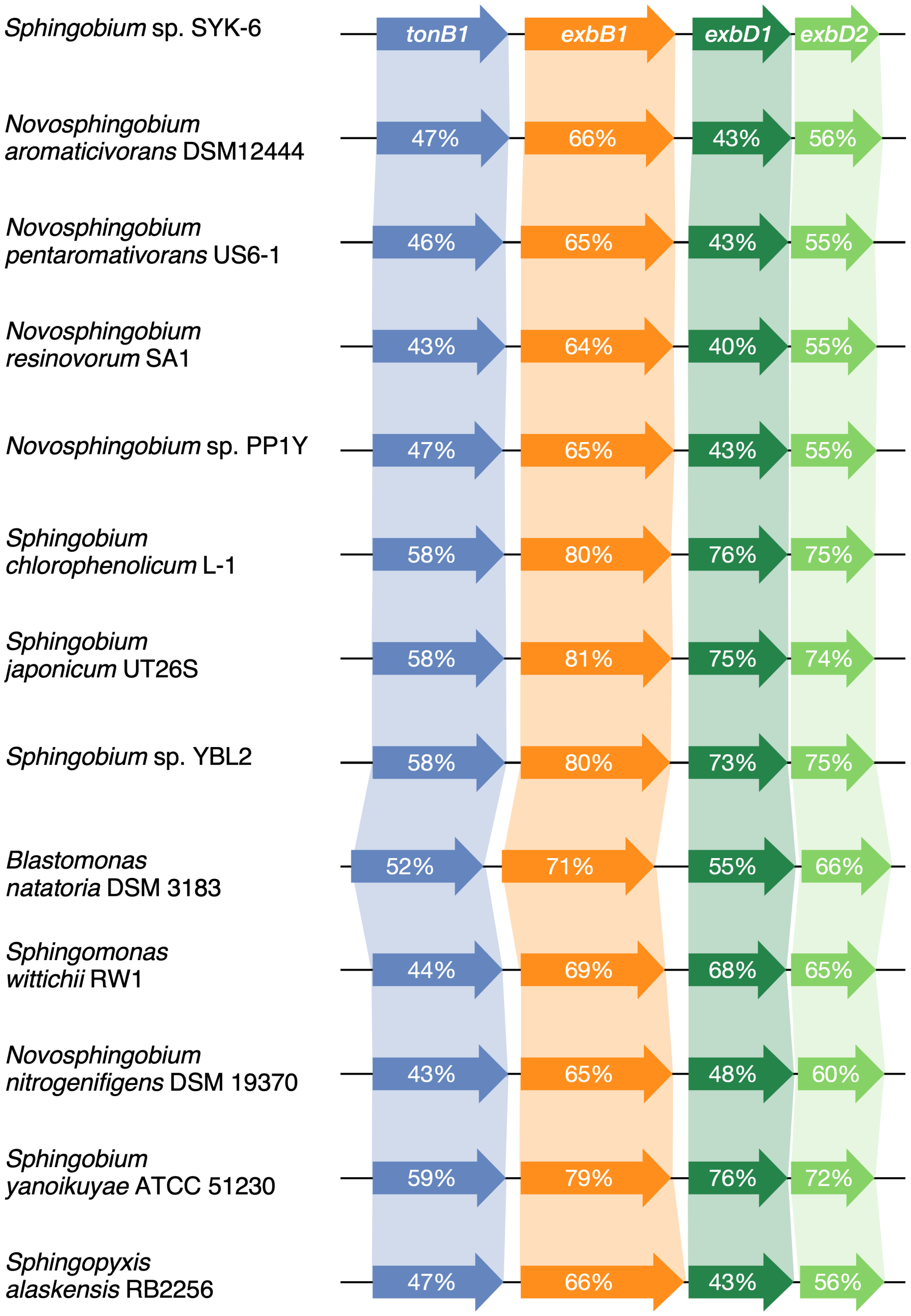

Fig. S12 Organization of the Sphingomonadaceae genes showing similarity with the *tonB1* operon genes of *Sphingobium* sp. SYK-6. Accession numbers of the genes are shown in Table S1. This figure was generated using Canvas X Draw version 7.0.2. (https://www.canvasgfx.com/products/canvas-x-draw).

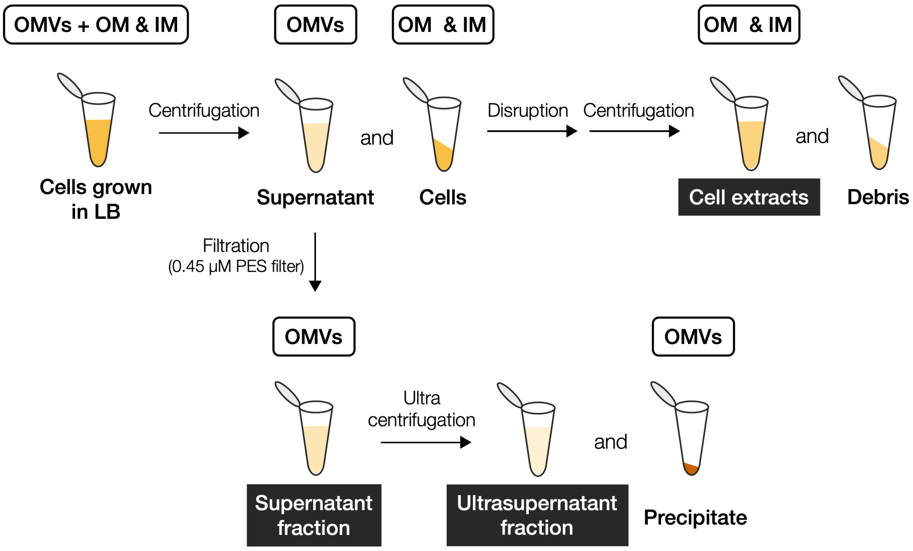

Fig. S13 Scheme of sample preparation for western blot analysis in Fig. 7. The supernatant fraction, ultrasupernatant fraction, and cell extracts were analyzed by western blotting. Details are given in the Methods. OMVs, outer membrane vesicles; OM, outer membrane; IM, inner membrane. This figure was generated using Canvas X Draw version 7.0.2. (https://www.canvasgfx.com/products/canvas-x-draw).

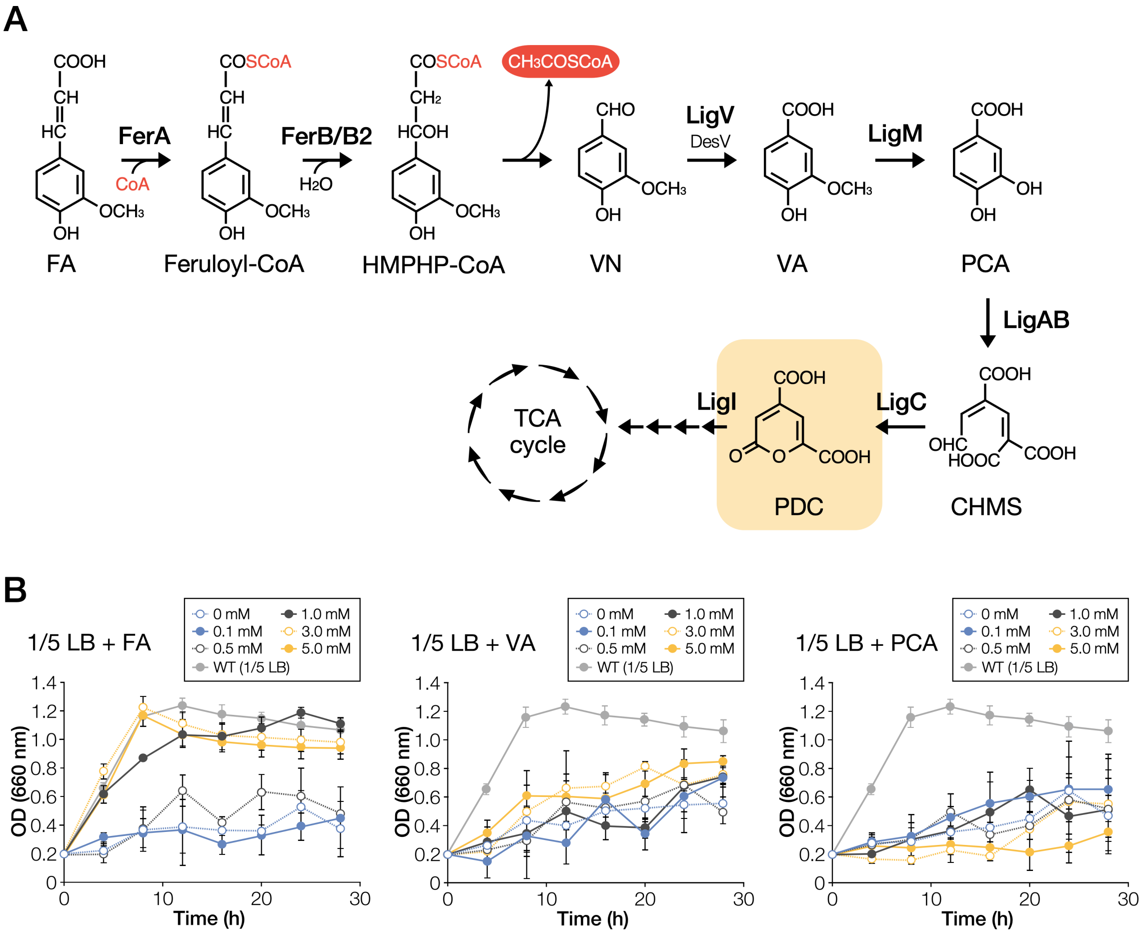

Fig. S14 Addition of FA markedly improved the growth of Δ*exbD3*/*tolR ligI* in LB. (A) Catabolic pathway of ferulate (FA) in SYK-6. Enzymes: FerA, feruloyl-CoA synthetase; FerB and FerB2, feruloyl-CoA hydratase/lyase; LigV, VN dehydrogenase; DesV, SN dehydrogenase; LigM, VA/3-*O*-methylgallate *O*-demethylase; LigA and B, small and large subunits of PCA 4,5-dioxygenase; LigC, CHMS dehydrogenase; LigI, PDC hydrolase. Compounds: HMPHP-CoA, 4-hydroxy-3-methoxyphenyl-β-hydroxypropionyl-CoA; CHMS, 4-carboxy-2-hydroxymuconate-6-semialdehyde; PDC, 2-pyrone-4,6-dicarboxylate. (B) Growth of Δ*exbD3*/*tolR ligI* cells in diluted LB with or without 0.1–5.0 mM FA, VA, or PCA. The growth of wild type cells in diluted LB shown in Fig. 3 is also indicated. Cell growth was monitored by measuring the OD_{660}. Each value is the average ± the standard deviation of three independent experiments. This figure was generated using Canvas X Draw version 7.0.2. (https://www.canvasgfx.com/products/canvas-x-draw).

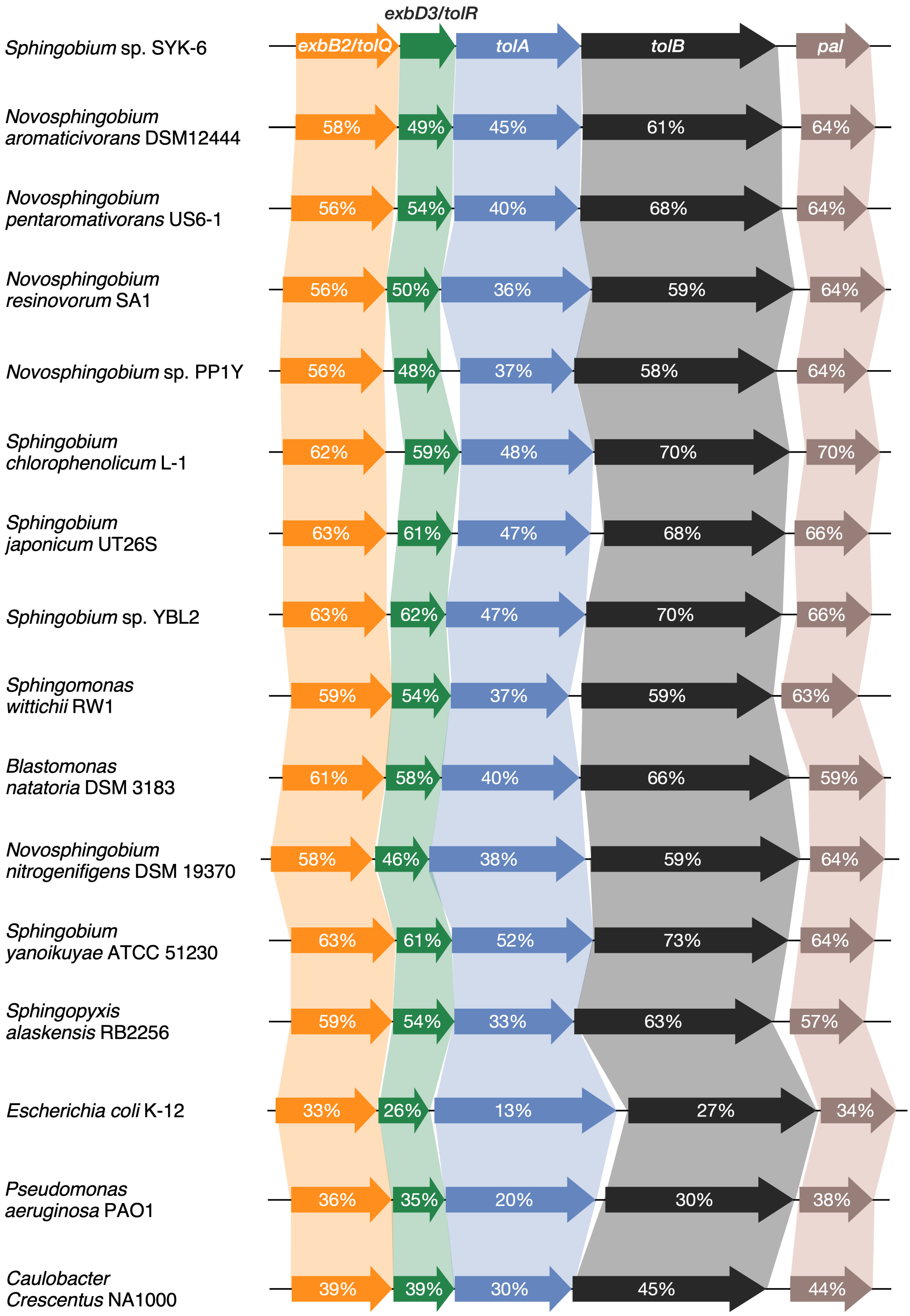

Fig. S15 Organization of the Sphingomonadaceae genes showing similarity with the Tol-Pal system genes of *Sphingobium* sp. SYK-6. Accession numbers of the genes are shown in Table S2. This figure was generated using Canvas X Draw version 7.0.2. (https://www.canvasgfx.com/products/canvas-x-draw).

A

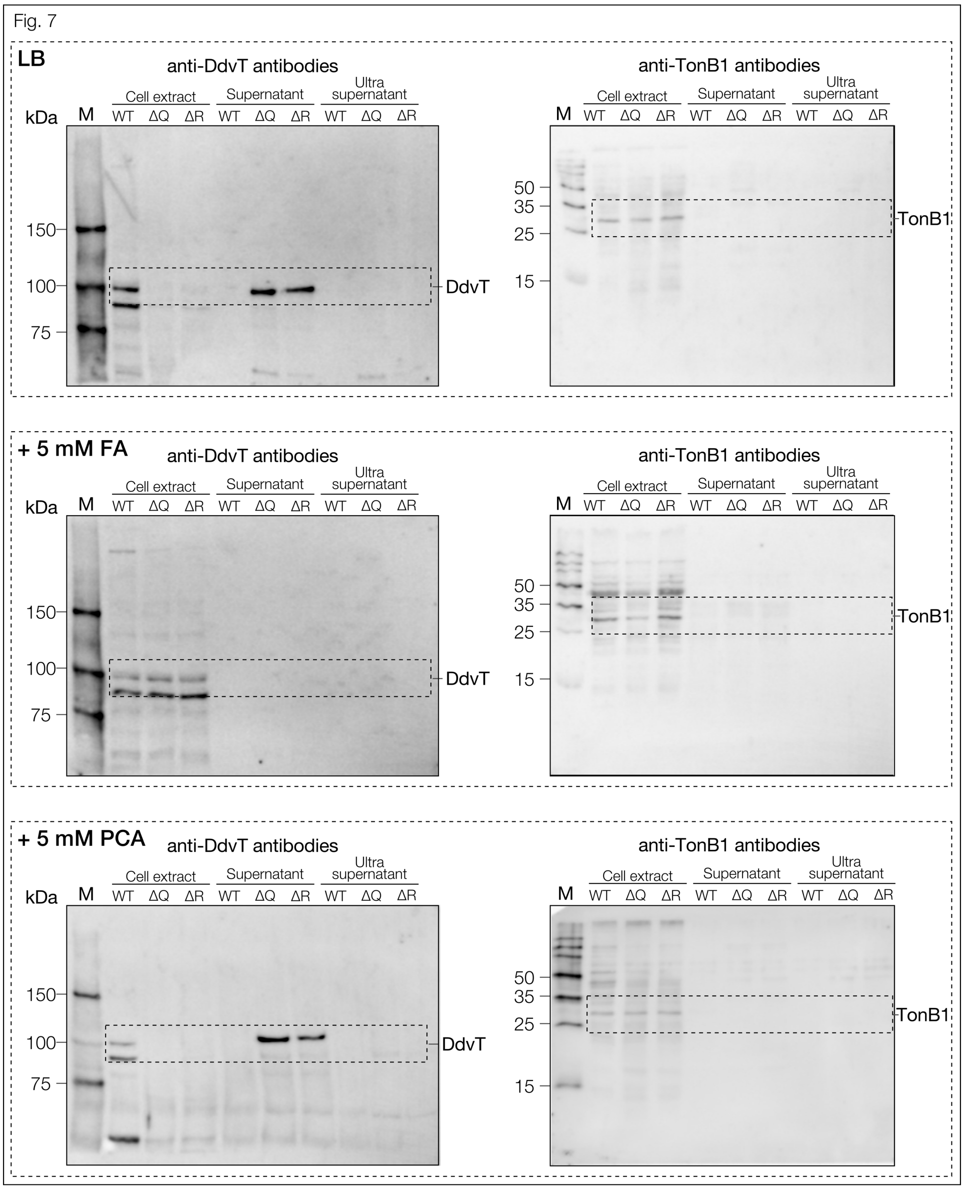

B

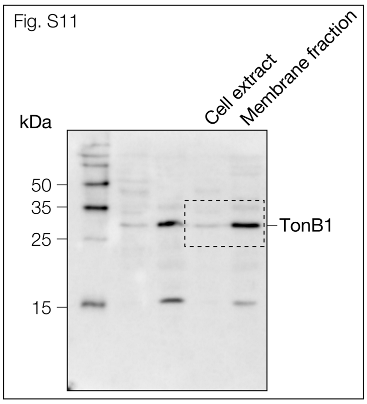

Fig. S16 Uncropped western blot images shown in Fig. 7 and Fig. S11. This figure was generated using

Canvas X Draw version 7.0.2. (https://www.canvasgfx.com/products/canvas-x-draw).

References

1 Johnson, M. *et al.* NCBI BLAST: a better web interface. *Nucleic Acids Res.* **36**, W5-9, doi:10.1093/nar/gkn201 (2008).

2 Katayama Y, N. S., Nakamura M, Yano K, Yamasaki M, Morohoshi N, Haraguchi T. Cloning and expression of *Pseudomonas paucimobilis* SYK-6 genes involved in the degradation of vanillate and protocatechuate in *P. putida*. *Mokuzai Gakkaishi* **33**, 77-79 (1987).

3 Fujita, M. *et al.* A TonB-dependent receptor constitutes the outer membrane transport system for a lignin-derived aromatic compound. *Commun. Biol.* **2**, 432, doi:10.1038/s42003-019-0676-z (2019).

4 Bolivar, F. & Backman, K. Plasmids of *Escherichia coli* as cloning vectors. *Methods Enzymol.* **68**, 245-267 (1979).

5 Figurski, D. H. & Helinski, D. R. Replication of an origin-containing derivative of plasmid RK2 dependent on a plasmid function provided in *trans*. *Proc. Natl. Acad. Sci. USA* **76**, 1648-1652, doi:10.1073/pnas.76.4.1648 (1979).

6 Blatny, J. M., Brautaset, T., Winther-Larsen, H. C., Karunakaran, P. & Valla, S. Improved broad-host-range RK2 vectors useful for high and low regulated gene expression levels in Gram-negative bacteria. *Plasmid* **38**, 35-51, doi:10.1006/plas.1997.1294 (1997).

7 Kaczmarczyk, A., Vorholt, J. A. & Francez-Charlot, A. Markerless gene deletion system for Sphingomonads. *Appl. Environ. Microbiol.* **78**, 3774-3777, doi:10.1128/AEM.07347-11 (2012).

8 Silva-Rocha, R. *et al.* The Standard European Vector Architecture (SEVA): a coherent platform for the analysis and deployment of complex prokaryotic phenotypes. *Nucleic Acids Res.* **41**, D666-675, doi:10.1093/nar/gks1119 (2013).

9 Mori, K., Kamimura, N. & Masai, E. Identification of the protocatechuate transporter gene in *Sphingobium* sp. strain SYK-6 and effects of overexpression on production of a value-added metabolite. *Appl. Microbiol. Biotechnol.* **102**, 4807-4816, doi:10.1007/s00253-018-8988-3 (2018).

10 Mori, K., Niinuma, K., Fujita, M., Kamimura, N. & Masai, E. DdvK, a novel major facilitator superfamily transporter essential for 5,5'-dehydrodivanillate uptake by *Sphingobium* sp. strain SYK-6. *Appl. Environ. Microbiol.* **84**, doi:10.1128/AEM.01314-18 (2018).

11 Fujita, M. *et al.* Iron acquisition system of *Sphingobium* sp. strain SYK-6, a degrader of lignin-derived aromatic compounds. *Sci. Rep.* **10**, 12177, doi:10.1038/s41598-020-68984-2 (2020).

12 Celia, H., Noinaj, N. & Buchanan, S. K. Structure and stoichiometry of the Ton molecular motor. *Int. J. Mol. Sci.* **21**, E375, doi:10.3390/ijms21020375 (2020).

13 Sievers, F. *et al.* Fast, scalable generation of high-quality protein multiple sequence alignments using Clustal Omega. *Mol. Syst. Biol.* **7**, 539, doi:10.1038/msb.2011.75 (2011).
